# Successful Resolution of Preretinal Haemorrhage with Intravitreal Ranibizumab

**DOI:** 10.1155/2016/4164198

**Published:** 2016-10-09

**Authors:** Baharuddin Noorlaila, Embong Zunaina, Mohd-Noor Raja-Azmi

**Affiliations:** Department of Ophthalmology, School of Medical Sciences, Universiti Sains Malaysia, 16150 Kubang Kerian, Kelantan, Malaysia

## Abstract

We would like to report two cases of preretinal haemorrhage from two different aetiology courses of bleeding being treated with intravitreal ranibizumab and its outcome. Our first case was a 39-year-old man with a diagnosis of severe aplastic anaemia that presented with bilateral premacular haemorrhages in both eyes. His right eye vision was 6/45 and it was counting finger in the left eye. He was treated with intravitreal ranibizumab once to the right eye and twice to the left eye. Right eye showed complete resolution of premacular haemorrhage and minimal residual premacular haemorrhage in the left eye at 3 months after initial presentation. Our second case was a 32-year-old healthy teacher that presented with preretinal haemorrhage at superotemporal region extending to macular area in left eye secondary to valsalva retinopathy. Her left vision was counting finger. She was treated with single intravitreal ranibizumab to the left eye. There was significant reduction of premacular haemorrhage and her left eye vision improved to 6/6 at 10 weeks after injection. Both cases had favourable outcome with intravitreal ranibizumab and can be considered as nonsurgical treatment option in treating premacular haemorrhage.

## 1. Introduction

Preretinal or premacular haemorrhage is a condition where the bleeding is located anterior to the internal limiting membrane [1]. Valsalva retinopathy and Terson syndrome are the common cause of premacular haemorrhage. Other causes include shaken baby syndrome, age-related macular degeneration, blood disorders, and bleeding secondary to retinal macroaneurysm, diabetic retinopathy, hypertension, retinal artery, or vein occlusion and also secondary to trauma [2–4].

We would like to report two cases of spontaneous preretinal haemorrhage secondary to aplastic anaemia and valsalva retinopathy and they were successfully treated with intravitreal ranibizumab.

## 2. Case Report

### 2.1. Case 1

A 39-year-old Malay male, a navy officer newly diagnosed with aplastic anaemia which was confirmed by trephine bone marrow biopsy, was referred from army hospital to haematology unit for further management of aplastic anaemia. He presented with severe lethargy, palpitation, and gum bleeding for 2 weeks and bilateral painless blurring of vision for 2 days prior admission to army hospital. He was referred to our ophthalmology clinic by haematology team for further eye management. The poor vision was not associated with floaters, flashes of light, or any field defects. There was no history of taking traditional medications, heavy lifting, straining, or prolonged coughing. There was no preceding history of trauma or exposure to noxious chemicals or irradiation. Premorbidly he had good vision in both eyes. Laser therapy was delivered to the left eye while he was admitted to army hospital. However, there was no improvement of vision.

**Figure 1:**
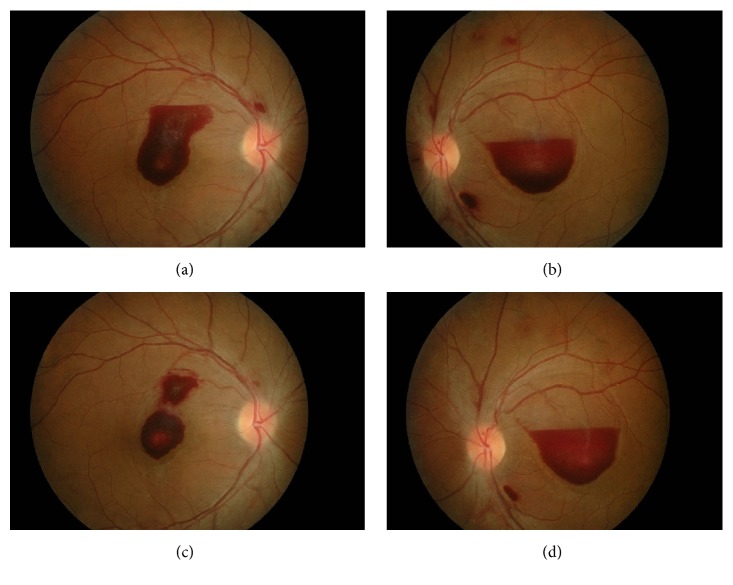
Case 1 at initial presentation and at 1 week after right intravitreal ranibizumab. Notes: (a) premacular haemorrhage at initial presentation in the right eye, (b) premacular haemorrhage at initial presentation in the left eye, (c) partial resolution of premacular haemorrhage in the right eye at 1 week after right intravitreal ranibizumab, and (d) minimal resolution of premacular haemorrhage in the left eye at 1 week after right intravitreal ranibizumab.

Ocular examination revealed visual acuity in the right eye was 6/45 and it was counting finger in the left eye. There was absence of relative afferent pupillary defect. Anterior segment examinations were unremarkable and the intraocular pressure measured 14 mmHg in both eyes. Fundus examination showed presence of premacular haemorrhage measuring two-disc diameter located at the macular area in both eyes (Figures 1(a) and 1(b)). There were no cotton wool spots, hard exudates, neovascularisation, and vitreous haemorrhage suggestive of any ischaemic condition. Multiple laser scars were present at peripheral retina in the left eye. Systemic examination showed no sign of bleeding tendency with absence of hepatosplenomegaly and lymphadenopathy.

Initial blood investigations upon admission to army hospital revealed low cell counts; haemoglobin levels were at 4.2 g/dL and platelets count was 8.0 × 10^9^/L. The haemoglobin levels were improved to 10.5 g/dL and platelets count was increased to 32 × 10^9^/L after receiving few packed cells and platelets transfusion upon presentation to haematology unit. Serum renal and clotting profiles were normal.

He was diagnosed with bilateral preretinal haemorrhage secondary to severe aplastic anaemia. Systemically, he was treated with antithymocyte globulin, cyclosporine, and prednisolone regime (ATGAM-CSA-Pred) and additional few blood transfusions by haematology team. However, his vision remains poor with slow resolution of his submacular haemorrhage while on systemic treatment.

**Figure 2:**
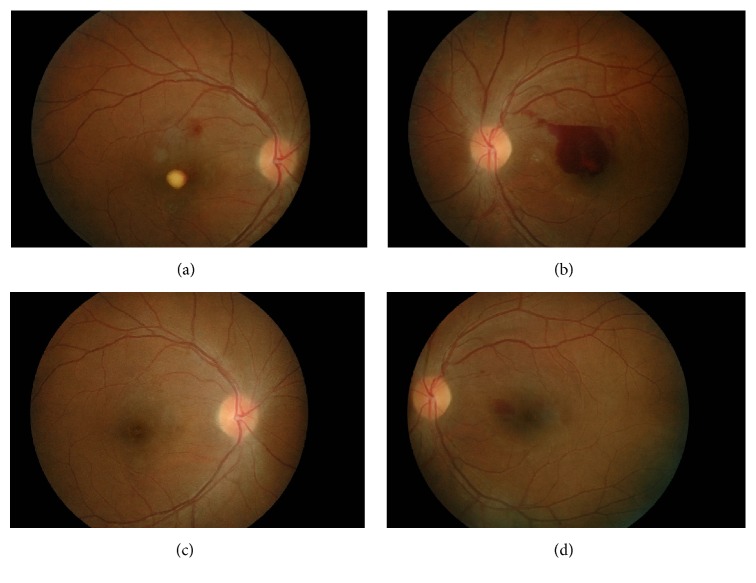
Case 1 at 6 weeks and 3 months after initial presentation. Notes: (a) premacular exudate in the right eye at 1 month after first left intravitreal ranibizumab (6 weeks after initial presentation), (b) partial resolution of premacular haemorrhage in the left eye at 1 month after first left intravitreal ranibizumab (6 weeks after initial presentation), (c) complete resolution of premacular haemorrhage in the right eye at 1 month after second left intravitreal ranibizumab (3 months after initial presentation), and (d) minimal residual premacular haemorrhage in the left eye at 1 month after second left intravitreal ranibizumab (3 months after initial presentation).

**Figure 3:**
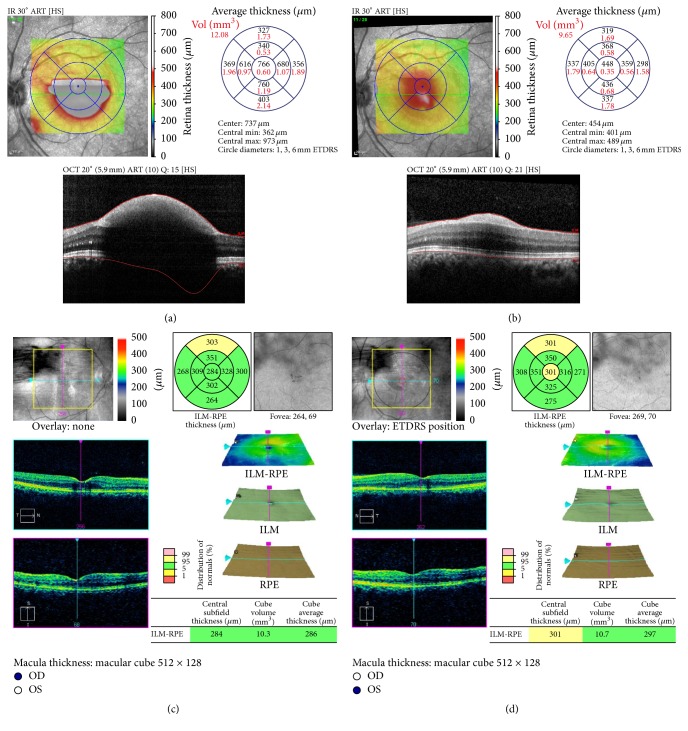
Case 1. Optical coherence tomography (OCT) at initial presentation and at 6 weeks and 3 months after initial presentation. Notes. (a) OCT showed premacular haemorrhage in the left eye at initial presentation. (b) OCT showed partial resolution of premacular haemorrhage in the left eye at 1 month after first left intravitreal ranibizumab (6 weeks after initial presentation). (c) Minimal residual premacular haemorrhage with normal retinal layer in the right eye (c) and left eye (d) at 3 months after initial presentation.

In view of slow resolution and the location of preretinal haemorrhage at the macular area, he was treated with intravitreal ranibizumab 0.5 mg to the right eye and conservative treatment in the left eye. The right eye vision improved to 6/15 with partial resolution of premacular haemorrhage (Figure 1(c)) at 1 week after procedure. However, there was no improvement of vision in the left eye with minimal resolution of premacular haemorrhage (Figure 1(d)). Since there was poor resolution of premacular haemorrhage in the left eye, intravitreal ranibizumab 0.5 mg was given to the left eye 1 week later. Follow-up at 1 month after left eye procedure (6 weeks after initial presentation) showed the right vision further improved to 6/9 with residual hard exudate at the fovea (Figure 2(a)). There was also improvement of vision in the left eye from counting finger to 6/24. However, there was only partial resolution of premacular haemorrhage (Figure 2(b)) in the left eye. Second intravitreal ranibizumab 0.5 mg was provided for the left eye at 6-week interval from the first left eye injection. At subsequent visit at 1 month after second left eye intravitreal ranibizumab (3 months after initial presentation), the visual acuity was 6/6 in both eyes and fundus examination showed complete resolution of premacular haemorrhage in the right eye (Figure 2(c)) and minimal residual premacular haemorrhage in the left eye (Figure 2(d)). Optical coherence tomography of the left eye showed resolution of premacular haemorrhage from initial presentation (Figure 3(a)) to partial resolution at 6 weeks after initial presentation (Figure 3(b)) and minimal residual premacular haemorrhage with normal retinal layer in both eyes at 3 months after initial presentation (Figures 3(c) and 3(d)).

### 2.2. Case 2

A 32-year-old Malay teacher presented with left eye sudden onset of reduced central vision upon waking up in the morning for 4 days. Her medical history was unremarkable except for few episodes of constipation and sneezing spells prior to onset. There was no preceding history of overt bleeding manifestations, ocular trauma, taking traditional medications, or exposure to noxious chemicals or irradiation.

**Figure 4:**
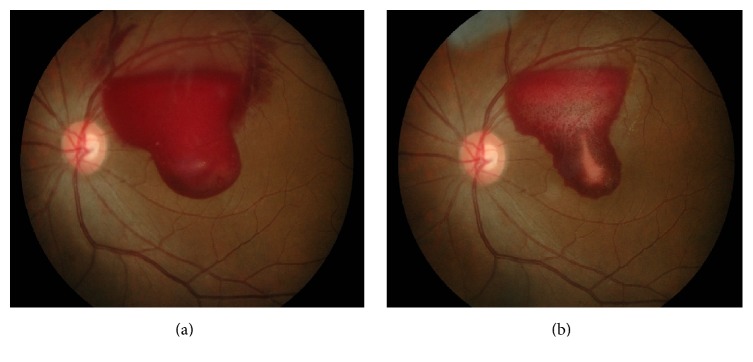
Case 2 at initial presentation and 2 months after initial presentation. Notes: (a) preretinal haemorrhage in the left eye at initial presentation and (b) minimal resolution of preretinal haemorrhage in the left eye at 2 months after initial presentation after conservative treatment.

Ocular examination revealed visual acuity in the right eye was 6/6 and counting finger in the left eye. There was absence of relative afferent pupillary defect. The anterior segment examinations were normal bilaterally with 15 mmHg intraocular pressure in both eyes. There was presence of preretinal haemorrhage at superotemporal region extending to macular area measuring 5 to 6 discs of diameter in the left eye (Figure 4(a)). There were no other retinal haemorrhages, cotton wool spots, hard exudates, neovascularisation, and vitreous haemorrhage suggestive of any ischaemic condition.

Blood pressure, coagulation profile, and erythrocyte sedimentation rate were normal with no thrombocytopenia. Additional tests for autoimmune diseases were negative.

**Figure 5:**
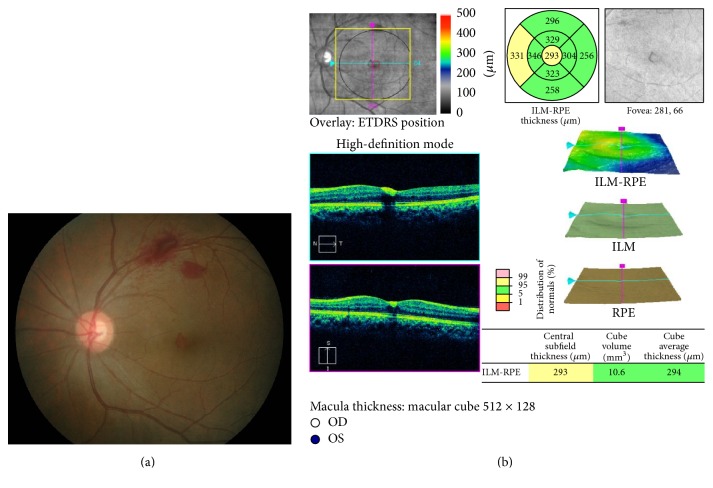
Case 2 at 1 month after intravitreal ranibizumab. Notes. (a) Resolution of preretinal haemorrhage in the left eye at 1 month after intravitreal ranibizumab. (b) Optical coherence tomography showed minimal residual preretinal haemorrhage at the fovea with normal retinal layer of the left eye at 1 month after intravitreal ranibizumab.

A clinical diagnosis of valsalva retinopathy was made. In view of young patient with no medical illness, initial management was just conservative treatment with instillation of topical nepafenac 8 hourly in the left eye. Her left vision remains poor with slow resolution of her preretinal haemorrhage at 2 months after initial presentation (Figure 4(b)). Treatment option was discussed with patient and the patient agreed on intravitreal ranibizumab. Single intravitreal ranibizumab 0.5 mg was given to the left eye at 2 months of the initial presentation. There was significant resolution of preretinal haemorrhage at 1 month following injection (Figure 5(a)) with 6/60 vision. Optical coherence tomography showed minimal residual preretinal haemorrhage at the fovea with normal retinal layer (Figure 5(b)). The left eye visual acuity returns back to premorbid 6/6 at 10 weeks after intravitreal ranibizumab with resolution of residual preretinal haemorrhage at the fovea.

## 3. Discussion

Aplastic anaemia is a blood disorder characterised by anaemia, leukopenia, and thrombocytopenia. It is a life-threatening condition but treatable. It can be inherited but it is more commonly acquired. Ocular manifestation is due to anaemic retinopathy. Anaemia causes diminished capillary oxygenation and subsequently causes increase in permeability of the blood vessel wall [4] and leads to extravasation of blood products [5]. Intraretinal haemorrhages and preretinal haemorrhages are the common ocular manifestation besides cotton wool spots. Other ocular manifestations are vitreous haemorrhages and optic disc oedema. The relationship between the severity of the retinopathy and the degree of anaemia showed a direct correlation [6].

Valsalva retinopathy is a form of retinopathy and premacular haemorrhage is a classical ocular manifestation. The mechanism of valsalva retinopathy is due to a sudden rise in intrathoracic or intra-abdominal pressure against closed glottis that may cause a rapid increase in venous pressure in the eye leading to spontaneous rupture of perifoveal retinal capillaries [7, 8]. Healthy young adults are a common age group that presented with valsalva retinopathy with history of valsalva-like maneuver such as heavy lifting, coughing, sneezing, vigorous sexual activity, bungee jumping, constipation, and also certain procedure that related to increase in intrathoracic or intra-abdominal pressure like fiberoptic gastroenteroscopy [9–12].

Preretinal haemorrhage may resolve spontaneously. Wan-Wei et al. [13] have reported in their case report that there was spontaneous recovery of preretinal haemorrhage and improvement of vision with oral prednisolone. However, in some patients, spontaneous resolution of preretinal haemorrhages may take months especially large and dense haemorrhage [14].

Retinal vasculature insult can lead to extensive tissue inflammation due to release of VEGF. Meanwhile intraretinal tissue migration and proliferation lead to formation of preretinal membrane, pigmentary macular changes [15, 16], and proliferative vitreoretinopathy [17]. Haemorrhage with prolonged contact releases biochemical components that may have the inclination to photoreceptor toxicity and toxic damage to the retina, resulting in permanent visual impairment [18]. These conditions are even more destructive in subinternal limiting membrane than in subhyaloidal haemorrhage [3]. For haemorrhage between the subinternal limiting membrane and subhyaloidal haemorrhage, the former tends to remain longer [3, 19].

There are several treatment options for the treatment of premacular haemorrhage such as pneumatic displacement of haemorrhage using either gas or tissue plasminogen activator, vitrectomy, and YAG laser hyaloidotomy [20–22].

Pneumatic displacement with or without recombinant tissue plasminogen activator is one of the treatment options for age-related macular degeneration associated with submacular haemorrhage [23]. The similar method of treatment has been successfully used to treat diabetic premacular haemorrhage [24]. However, shearing of the photoreceptor layers can occur if the technique of the procedure was not properly done and this can lead to poor visual outcome [25].

Vitrectomy is an invasive option that allows clearance of haemorrhage. However, there are multiple side effects that are associated with vitrectomy such as cataract, retinal breaks, retinal detachment, and endophthalmitis [1, 26].

Laser drainage related complications have been reported as epimacular membranes, macular hole formation, and retinal detachment [27].

Intravitreal ranibizumab is a prime treatment available for treating submacular haemorrhage that occurs in age-related macular degeneration, polypoidal choroidal vasculopathy, proliferative retinopathy, and trauma [28].

As far as we know, there have been no reported cases of premacular haemorrhage secondary to aplastic anaemia and valsalva retinopathy treated with ranibizumab. In our case report, the patients had satisfactory recovery of their vision and restoration of retinal architecture with intravitreal ranibizumab which is less invasive day care procedure. There was a dramatic visual recovery observed in both eyes at one month after ranibizumab injection. Although spontaneous resolution of haemorrhage can occur, anti-VEGF injection had fastened the resolution of premacular haemorrhage in both of our patients. Visual acuity improved to 6/6 in both eyes at 3/12 after injection with normal anatomical and functional recovery. Anti-inflammatory and antiangiogenesis properties of anti-VEGF were postulated to play an important role in the clearance of haemorrhage and also as antiscarring effect in our patients with premacular haemorrhage. Intravitreal anti-VEGF therapy blocks inflammatory cell infiltration and may serve as a treatment in retinal inflammation [29].

Intravitreal ranibizumab can be considered as nonsurgical treatment option in treating premacular haemorrhage with favourable outcome. The role of anti-VEGF in treating premacular haemorrhage needs further evaluation.
